# Содержание желтых пигментов в зерне твердой пшеницы (Triticum durum Desf.): биосинтез, генетический контроль, маркерная селекция

**DOI:** 10.18699/VJ20.642

**Published:** 2020-08

**Authors:** P.N. Malchikov, M.G. Myasnikova

**Affiliations:** Samara Federal Research Scientific Center of the Russian Academy of Sciences, Samara Scientific Research Agriculture Institute named after N.M. Tulaikov, Bezenchuk, Samara region, Russi; Samara Federal Research Scientific Center of the Russian Academy of Sciences, Samara Scientific Research Agriculture Institute named after N.M. Tulaikov, Bezenchuk, Samara region, Russi

**Keywords:** durum wheat, carotenoids, concentration pigment, yellow index, marker-assisted selection, твердая пшеница, каротиноиды, концентрация пигментов, индекс желтизны, маркерная селекция

## Abstract

Зерно с высоким содержанием каротиноидных пигментов ценится за ярко-желтый цвет пасты, производимой из него, и провитаминную (витамин А) и антиоксидантную активность пигментов. Цель настоя- щего обзора – обобщение современных знаний о биосинтезе и генетическом контроле накопления пигментов в зерне твердой пшеницы и оценка основных результатов исследований и селекции за последние двадцать лет за рубежом и в России. Признак «концентрация каротиноидных пигментов в зерне» (Ypc) относится к раз- ряду количественных. Тем не менее превалирование сильных аддитивных эффектов генов и высокая насле- дуемость способствовали значительному прогрессу в селекции по этому признаку. Методами молекулярного маркирования локусов количественных признаков (QTL), контролирующих синтез каротиноидных пигментов и значения индекса желтизны (IY), установлено их распределение по всем хромосомам генома твердой пшеницы. Основные генетические локусы, определяющие более 60 % варьирования признака, были картированы в хромосомах 7AL и 7BL. Вклад этих локусов связан с аллельными вариациями, влияющими на активность фермента фитоенсинтетазы (PSY). В других хромосомах были локализованы минорные генетические факторы, из которых наиболее значимы QTL, расположенные в хромосомах 3AS (ассоциирован с геном LCYE-ликопин- ε-циклаза) и 4ВS (аллель Lpx-B1.1c). При этом показано, что аллель Lpx-B1.1c вносит вклад в снижение актив- ности липоксигеназы, окисляющей каротиноиды в процессе изготовления конечных продуктов. Рассмотрены и обсуждены проблемы использования молекулярных маркеров в селекционных программах, нацеленных на увеличение концентрации пигментов в зерне и улучшение цветовых характеристик пасты.

## Abstract (en)

Grain with high contents of yellow pigments will add the natural bright-yellow colour to the paste, which unlike a paste with a high level of whiteness, are preferred by consumers. The provitamin activity (vitamin A) and antioxidant activity of the carotenoid pigment increase the biological and nutritional value of the grain with high contents of these pigments. The purpose of this review is to summarize modern knowledge about the biosynthesis and genetic control of pigment accumulation in durum wheat and to assess the main results of research and selection over the past 20 years abroad and in Russia. The trait “concentration carotenoid pigment in grain” (Ypc) is quantitative. However, the prevalence of strong additive gene effects and high heritability have contributed to significant progress in breeding for this trait. Molecular labeling of quantitative trait loci (QTL) that control the synthesis of the carotenoid pigment and the yellowness index (YI) found that they are distributed across all chromosomes of the durum wheat genome. The main QTLs, which determine 60 % of the variation of the trait, were mapped to 7AL and 7BL chromosome. The contribution of these QTLs is associated with allelic variations that control the activity of phytoene synthase (PSY). QTLs with minor effects found on the remaining chromosomes are also reliably mapped using molecular markers. As confirmed in a number of experiments, most of them are QTLs located on 3AS (linked to the LCYE (lycopene ε-cyclase) allele and on 4BS (the LpxB1.1c gene). It has been shown that the LpxB1.1c allele contributes to a decrease in the activity of lipoxygenase, which oxidases carotenoids during the production of end products. This review considered and discusses the problems of molecular markers in breeding programs to increase the concentration of pigments in the grain and improve the color characteristics of the paste.

## Введение

Твердая пшеница (Triticum durum Desf.) ежегодно вы- ращивается во всем мире на площади около 17.0 млн га. Производство зерна колеблется по годам от 32 до 42 млн тонн. Продукты из твердой пшеницы используются почти во всех странах, но основными регионами потребления являются страны Средиземноморского бассейна, где зер- но твердой пшеницы применяется в качестве сырья для различных продуктов, в основном пасты и круп кус-кус и булгур (Kabbaj et al., 2017). Содержание и структура белка (качество клейковины), а также цвет изделий (макароны, крупа) – наиболее ценные для пищевой, технологической промышленности и на рынке конечных продуктов в этих регионах (Sisson, 2008; Mazzeo et al., 2017). Желтый цвет определяется накоплением каротиноидов в эндосперме, обладающих провитаминными и антиоксидантными свойствами. В процессе размола зерна, замеса теста и изго- товления макарон происходит окисление кислородом воз- духа полиненасыщенных жирных кислот, каротиноидов, фенолов, что приводит к накоплению бурого (коричне- вого) пигмента. Окисление катализируется ферментами: липоксигеназой, полифенолоксидазой, пероксидазой, альдегидоксидазой (N’Diaye et al., 2017). Наличие спексов (темных вкраплений) также может снижать уровень желтизны семолины и макаронных изделий (Васильчук и др., 2009).

Результаты изучения наследования всего комплекса желтых пигментов в зерне твердой пшеницы показали, что оно носит количественный характер с высокими зна- чениями коэффициента наследуемости и преобладанием аддитивных эффектов генов (Clarke еt al.,1998; Borelli et al., 1999; Digesù et al., 2009; Blanco et al., 2011; Roncallo et al., 2012; Schulthess et al., 2013). В связи с этим право- мерно предположение о том, что этот признак удобен для молекулярного маркирования соответствующих QTL и их картирования на хромосомах.

## Биосинтез, содержание и распределение каротиноидов в зерновке твердой пшеницы

Средняя концентрация каротиноидов в зерне твердой пшеницы составляет 6.2 ± 0.13 мг/кг в сухом весе (Brandolini et al., 2015) с варьированием, в зависимости от сорта и условий среды (год, пункт), от 2.8 до 12.3 мг/кг (Colasuonno et al., 2017a). По мнению Н.С. Васильчука с коллегами (2009), в Поволжье при низкой активности окислительных ферментов достаточно иметь зерно с кон- центрацией 4.5 мг/кг для получения макарон золотисто- желтого цвета. Каротиноиды – не единственные желтые пигменты в зерне пшеницы и других злаков. Сравнивая общую концентрацию каротиноидов, определенную ме- тодом высокоэффективной жидкостной хромотографии (ВЭЖХ), с общим содержанием пигментов, A.M. Digesù с коллегами (2009) показали, что доля каротиноидов у культивируемых и диких тетраплоидных видов пше- ницы составила 33.2 % от общего количества желтых пигментов. По результатам своих исследований A. Blanco с колегами (2011) также сообщили о доле каротиноидов, составившей 37 % в общем объеме желтых пигментов, что означает наличие в экстрактах твердой пшеницы неизвест- ных желтых пигментов, поглощающих свет при 435 нм. По данным (Fu et al., 2017), полученным при изучении канадских сортов твердой пшеницы Navigator и Strongfield, фенольные соединения могут вносить существенный вклад в степень желтизны при экстракции пигментов. Brandolini с коллегами (2008) пришли к выводу, что, хотя каротиноиды являются наиболее важными пигментами при определении желтого цвета пшеничной муки и мака- рон, их точное измерение может быть достигнуто только с помощью ВЭЖХ анализа. Тем не менее среди желтых пигментов именно по каротиноидам имеется более под- робная научная информация.

К каротиноидам относятся пигменты – каротины и ксантофиллы. Химически они представляют собой изо- преноидные углеводороды, содержащие 40 углеродных атомов (Кретович, 1986). Биосинтез каротиноидов тща- тельно исследован на различных растениях – арабидоп- сисе, рисе, кукурузе, перце, томатах, апельсине и других культурах (Colasuonno et al., 2017a; Rodrigues-Concepcion et al., 2018; Sun et al., 2018).

В зерне твердой пшеницы представлен широкий набор каротиноидных пигментов: лютеин, β-каротин, зеаксантин, β-криптоксантин, β-апокаротенал, антраксантин, тараксантин (лютеин-5,6-эпоксид), авоксантин и тритикоксантин. Каротины α и β в основном находятся в за- родыше, превалирующий в зерне среди каротиноидов лютеин (86–94 %) одинаково распределен по слоям и частям зерновки (Digesù et al., 2009). Его доля в семолине при размоле зерна твердой пшеницы составляет 83 %, во фракциях отрубей – 75 % (Fu et al., 2017). Установлено, что характер распределения желтых пигментов по фракциям, выделяемым в процессе размола, варьирует, в зависимо- сти от генотипа. Концентрация лютеина в эндосперме сорта Navigator была выше, чем в отрубях, в то же время у сорта Strongfield наблюдалась обратная закономерность (Fu et al., 2017). В процессе помола и получения крупки (семолины) концентрация большинства каротиноидных пигментов уменьшается, что связано с увеличением кон- такта измельченных частиц зерна с кислородом воздуха и активностью ферментов. Лютеин и зеаксантин имеют более высокую стабильность при помоле и изготовлении конечных продуктов, по сравнению с другими каротиноидами (Kean et al., 2011).

Накопление каротинов в зерне, особенно β-каротина, обуславливает изменение интенсивности окраски семоли- ны от желто-оранжевого до красноватого оттенка. Ксан- тофиллы обеспечивают желто-оранжевую окраску крупки и макарон. Схема биосинтеза каротиноидов показана на рисунке. Исходным веществом для биохимического син- теза каротинов служит 5-углеродный (С-5) изопреноид – изопентилпирофосфат. Конденсация этого изопреноида представляет собой основу для образования геранил- геранилпирофосфата (С-20). В результате соединения двух молекул геранилгеранилпирофосфата при участии фермента фитоенсинтетазы образуется фитоен (С-40) – первое промежуточное вещество в биосинтезе каротинов. Этот этап является ключевым – скорость биосинтеза и на- копления фитоена влияет на весь пул каротиноидов (Cazzonelli, Pogson, 2010; Ke et al., 2019). Фитоен в результате десатурации под действием ферментов фитоендесатуразы (PDS), z-каротиндесатуразы (ZDS), каротинизомеразы (CRTISO) и последовательного удаления четырех атомов водорода превращается в ликопин. Ликопин – каротиноид, определяющий красную и оранжевую окраску плодов, – исходное вещество для синтеза α-каротина/лютеина (класс ксантофиллов) – главного каротиноида зерна твердой пшеницы и β-каротина/зеаксантина (класс ксантофил- лов) – главного компонента каротиноидных пигментов в зерне кукурузы (Zhang, Dubcovsky, 2008). Дальнейшее гидроксилирование α-каротина приводит к образованию желтого зейоксантина и лютеина. Трансформация β-каротина продуцирует образование β-криптоксантина, зеа- ксантина, антраксантина, виолаксантина и неоксантина. Эти реакции катализируются двумя негемовыми β-каротингидроксилазами (ВСН1 и ВСН2) и двумя гемгидроксилазами (CYP97A и CYP97C) соответственно (Sun et al., 2018). Последняя фаза биосинтеза каротиноидов, катализируемая неоксантиноксидазой (NXS), заключается в превращении виолаксантина в неоксантин. Окисление виолаксантина и неоксантина приводит к образованию ксантоксина, превращаемого в растительный гормон – абсцизовую кислоту (ABA), которая способствует регу- лярному и сбалансированному накоплению пигментов в растениях и формированию устойчивости к абиотическим стрессам (Al-Babilli, Bowmeester, 2015; Nisar et al., 2015). Еще одна ветвь трансформации β-каротина представляет превращение его под действием ферментов диоксигеназ- ной группы (CCD7, CCD8, CYP711A1) в стриголактоны – ингредиенты гормональной природы, регулирующие развитие и ростовые процессы растений (Colasuonno et al., 2019).

**Fig. 1.**
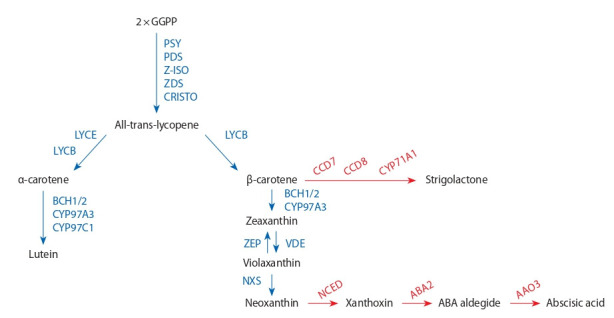
Biochemical reactions of the carotenoid pathway (Colasuonno et al., 2019). The main components of the biosynthetic pathway are shown in black; all enzymes involved in carotenoid synthesis, in blue; enzymes of the dioxygenase group involved in carotenoid metabolism in growing plants under stress and accumulating hormone-like ingredients – abscisic acid and strigolactones – in red.

## Методы определения общего и компонентного состава каротиноидных пигментов в зерне

Содержание пигментов в зерне, крупке и макаронах от- носится к сложным признакам. Для общего и компонент- ного определения их содержания существует несколько мотодов. Эталонные методы для определения общего содержания каротиноидов – стандартный метод 152 (ICC Method 152, 1990) Международной ассоциации науки и техники о зерне (ICC) и международный официальный метод (ААСС 14-50.01 (AACC International, 2013). Эти две процедуры основаны на экстракции всех пигментов в водонасыщенном н-бутаноле с последующим спектрофотометрическим количественным определением оптиче- ской плотности спиртового экстракта при 435.5 нм (длина волны максимального поглощения лютеина, доминирующего каротиноида в твердой пшенице). В качестве альтер- нативы концентрации для характеристики насыщенно- сти пигментами зерна применяется индекс желтизны (YI) на основе количественного определения коэффициента отражения света. Хроматограф Minolta CR-300 (Konica Minolta Pty Ltd, Macquarie Park, NSW), оснащенный им- пульсной ксеноновой дуговой лампой, – наиболее часто используемый инструмент для анализа индекса желтизны. Этот прибор позволяет определять коэффициенты индекса желтизны и коричневатости (100-L) семолины и макарон. В России в некоторых селекционных центрах для опреде- ления индекса желтизны применяется прибор Specol 10 по методологии, предложенной Н.С. Васильчуком (2001). Методы быстродействующей жидкостной хромотографии высокого давления (HPLC) с применением инфракрасной спектроскопии позволяют определить химический состав каротиноидных пигментов и измерить количество каждого компонента в крупке и пасте (Brandolini et al., 2008; Fu et al., 2017).

## Картирование локусов, контролирующих синтез пигментов

Концентрация пигментов в зерне твердой пшеницы кон- тролируется различными генами с аддитивными эффекта- ми и зависит от условий внешней среды (Васильчук и др., 2009; Мальчиков, 2009; Schulthess, Schwember, 2013; Гапо- нов и др., 2018; Мясникова и др., 2019). Систематическое сортоизучение в различных экологических условиях дает необходимую информацию о свойствах сортов и наличии у них соответствующих QTL. Достоверные, значительные и стабильные различия между генотипами по величинам YPC (Yellow Pigment Concentration) и YI (Yellowness Index) свидетельствуют о функционировании локусов количе- ственного признака. Для маркирования и локализации QTL используют рекомбинантно-инбредные линии (RIL), созданные от двуродительских скрещиваний контрастных по величине признака сортов и отобранные в поколениях (от одного парного скрещивания) из беккроссных или дигаплоидных популяций (Elouafi еt аl., 2001; Pozniak et al., 2007; Singh et al., 2009; Colasuonno et al., 2014). В настоящее время для этих целей используется метод полногеномного генотипирования для поиска корреляций между генотипами и фенотипами в наборах селекционных линий, образцов генетических коллекций и выявления аллельных вариантов молекулярных маркеров и функцио- нальных генов (Vargas et al., 2016; Colasuonno et al., 2017a; Fiedler et al., 2017). В последнее десятилетие этот подход получил распространение благодаря наличию большого количества ДНК-маркеров, равномерно распределенных в геноме, полученных на основе однонуклеотиного по- лиморфизма (SNP) и совершенствования статистических инструментов (Чесноков, Артемьева, 2011; Maccaferri et al., 2011; Wang et al., 2014; Sehgal, Dreisigacker, 2019). Картирование QTL предполагает хромосомную локали- зацию локуса в геноме по результатам оценки признаков, полученных в нескольких экспериментах.

Воспроизводимость результатов может зависеть от влияния на признак в исследуемой картирующей попу- ляции многочисленных генов с аддитивными эффектами, родительских компонентов, взаимодействия генотип–сре- да, числа используемых маркеров и способа измерения каротиноидов. В настоящее время для YPC и YI иденти- фицирован 81 QTL, включая синглетоны и кластеры QTL, которые распределены по всем хромосомам (Colasuonno et al., 2019). Локализация некоторых QTL была подтверждена с использованием нескольких картирующих популя- ций, что указывает на присутствие стабильных аллелей, влияющих на улучшение цвета и пищевой ценности зерна твердой пшеницы. Стабильные QTL были обнаружены в хромосомах 1А, 1В, 2А, 2В, 3В, 4А, 6А, 6В, 7А, 7В (Parker et al., 1998; Hessler et al., 2002; Patil et al., 2008; Zhang et al., 2009; Pozniak et al., 2012).

По эффектам на фенотипическую вариабельность признаков QTL распределяются на группы с сильным (40 %), средним (10–40 %) и незначительным (< 10 %) влиянием (Colasuonno et al., 2019). Главные локусы с сильным влиянием на YPC и YI были картированы в хромосоме 7АL и дистальной области 7BL (Elouafi et al., 2001; Pozniak et al., 2007; Patil et al., 2008; Zhang, Dubcovsky, 2008). В частности, в длинном плече хромосомы 7A были идентифицированы два QTL с противоположными эффектами. Локус с негативным эффектом по отношению к признакам YPC и YI был ассоциирован с аллельными вариациями гена альдегидоксидазы (АО) – фермента, катализирующего деградацию каротиноидов в результа- те их окисления (Colasuonno et al., 2014, 2017b). Второй (QTL-73), расположенный в области локализации гена Psy-1, оказывал положительный эффект (до 60 % вариации) на признак YPC. Аналогичные ассоциации QTL и YPC установлены в хромосоме 7BL – негативный эффект одного локуса на фенотипическое проявление YPC составлял 29 %, позитивное влияние второго локуса – 52 % (He et al., 2008, 2009; Zhang, Dubcovsky, 2008). В публи- кации (N’Diaye et al., 2017) сообщается о картировании YPC и Yi методом конструирования гаплотипных блоков. Гаплотипные блоки формируются с применением насы- щенных молекулярных SNP карт. При этом SNP, распо- ложенные в хромосоме в пределах 5.3 сМ, группируют в один гаплотипный блок, определяющий один локус, ко- торый обозначается префиксом “hap” с указанием номера хромосомы и порядкового номера локуса на хромосоме. Этот подход подтвердил существование высокозначимого QTL (hap_7A_32, маркер Tdurum_conting 54832_139) в хромосоме 7АL, объясняющего 35.6 % фенотипической дисперсии общего пигмента и индекса желтизны в семо- лине твердой пшеницы и связанного с локусом Psy-A1. Использование подобного метода подтвердило достовер- ную значимость QTL в хромосомах 2A (hap_2A_18), 7B (hap_7B_36) и 4B (hap_4B_6).

Незначительные по эффектам QTL обнаружены в хро- мосомах 3А (Parker et al., 1998), 4A и 5A (Hessler et al., 2002), 2A, 4B и 6В (Pozniak et al., 2007), 4B и 6B (Zhang, Dubcovsky, 2008), 1А, 3В и 5В (Patil et al., 2008), 3B и 5B (Howitt et al., 2009), 1A, 1B, 3B и 4А (Zhang et al., 2009).

## Гены, участвующие в биосинтезе каротиноидов и их окислении в процессе изготовления конечных продуктов

В настоящее время получена информация о генах-канди- датах, расположенных в регионах локализации локусов со значимым влиянием на признаки YPC и YI. В данном слу- чае к ним относятся гены, контролирующие активность основных ферментов биосинтеза каротиноидов пшеницы, и катаболические гены, вызывающие деградацию каро- тиноидов. Количественные различия генотипов по на коплению пигментов связаны с аллельным разнообра- зием генов PSY – фитоенсинтетазы (Pozniak et al., 2007; He et al., 2008; Dibari et al., 2012; Colasuonno et al., 2014; Campos et al., 2016), LCYE – ликопин-ε-циклазы (Howitt et al., 2009; Crawford, Francki, 2013), LCYB – ликопин-β- циклазы (Zeng et al., 2015), HYD – β-гидроксилазы (Qin et al., 2012), PDS (фитоендесатуразы) и ZDS – каротин- десатуразы (Cong et al., 2010). Катаболические гены контролируют активность альдегидоксидазы – АО (Colasuonno et al., 2017b), полифенолоксидазы – РРО (Watanabe et al., 2004, 2006; Si et al., 2012), липоксигеназы – LOX или Lpx (DeSimone еt al., 2010; Randhawa et al., 2013) и пероксида- зы – PER (Ficco et al., 2014), снижающих концентрацию пигментов и потребительские качества конечных продук- тов. Наиболее значимые гены, их аллельные варианты и маркеры обсуждаются в тексте и представлены в табл. 1.

**Table 1.**
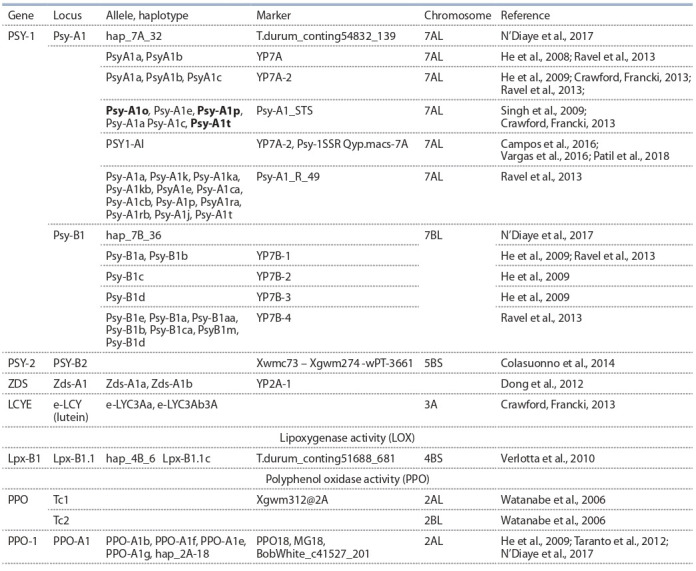
Genes and their markers associated with the concentration of yellow pigments in grains and processed products located in wheat genomes A and B

Как уже отмечено, ключевым ферментом в сложной це- почке биосинтеза каротиноидов является PSY. Известны три различных гена, кодирующих активность этого фер- мента: PSY 1, PSY 2, PSY 3, картированные в гомеологич- ных хромосомах 7-, 5- и 3-й групп соответственно (Dibari et al., 2012). Ген PSY-1 был локализован в хромосомах 7А и 7В твердой пшеницы. Обнаружено, что ген PSY-B1, расположенный в хромосоме 7В, сегрегирует совместно с QTL, ассоциированным с каротиноидным пулом, с измен- чивостью YI и YPC от низкого уровня (10 %) до среднего (10–30 %). Ген PSY-A1, расположенный в хромосоме 7А, ведет себя как кодоминантный маркер, объясняет фено- типическую изменчивость, в зависимости от генофона, в диапазоне от среднего (10–30 %) до высокого (30–50 %) и очень высокого (> 50 %) уровня (Colasuonno et al., 2014). В целом влияние альтернативных аллелей PSY-A1, по- видимому, является наиболее важным в изменчивости концентрации пигментов и индекса желтизны крупки. Этот вывод основан на результатах изучения различных популяций твердой пшеницы (Campos et al., 2016; Vargas et al., 2016; Patil et al., 2018). Ген PSY-B2, локализованный в 5ВS, не оказывал существенного влияния на концен- трацию каротиноидов в зерне и крупке (Colasuonno et al., 2014). Локализованный в длинном плече хромосомы 5В ген PSY-B3 повышал экспрессию в листьях и корнях в условиях абиотического стресса (засуха, засоление) и был связан с увеличением пула абсцизовой кислоты (Dibari et al., 2012). Гены, контролирующие активность других ферментов, также имеют значимое влияние на концентрацию пигментов в зерне и конечных продуктах. Так, ген ликопин-ε-циклазы (LCYE), ассоциированный с QTL на хромосоме 3А, играет определяющую роль в процессах накопления лютеина – главного каротиноида зерна твердой пшеницы (Howitt et al., 2009). Ген z-каротиндесатуразы (ZDS), маркируемый кодоминантным функциональным маркером YP2A-1 на основе полимор- физма двух аллелей, расположенный на хромосоме 2А, объяснял 11.3 % фенотипической дисперсии YPC и IY в популяции дигаплоидных линий (Dong et al., 2012).

Наиболее четко выражены гены-кандидаты, участвующие в катаболическом пути окисления каротиноидов. Липоксигеназа (Lpx) у растений продуцирует активные формы кислорода, приводящие к деградации каротинои- дов и обесцвечиванию конечных продуктов, получаемых из зерна твердой пшеницы (Borrelli et al., 2003). У твердой пшеницы существуют разные гены Lpx (Borrelli, Trono, 2016). На стадии зрелого зерна у сортов с контрастной активностью (окислительной способностью) была уста- новлена различная степень транскрипции генов Lpx-1 и Lpx-3, в то время как транскрипты Lpx-2 на стадии зрелого зерна отсутствовали (De Simone et al., 2010). Локус Lpx-B1 расположен на коротком плече хромосомы 4B, в кото- ром обнаружены три тесносцепленных гена: Lpx-B1.1, Lpx-B1.2 и Lpx-B1.3. Ген Lpx-B1.1 представлен тремя аллелями – Lpx-B1.1a, Lpx-B1.1b, Lpx-B1.1c (Hessler et al., 2002; Carrera et al., 2007; Verlotta et al., 2010). Анализ QTL у твердой пшеницы показал, что 35–54.0 % вариации активности Lpx объясняется Lpx-B1. Аллель Lpx-B1.1c отличается делецией в нуклеотидной последовательности от второго интрона до последнего экзона (Carrera et al., 2007). Этот аллель коррелирует с высоким уровнем жел- тизны и относительно слабой деградацией пигментов в макаронных изделиях (Carrera et al., 2007; Verlotta et al., 2010). Изучение коллекции твердой пшеницы с включе- нием в нее ландрасов и современных сортов позволило идентифицировать три гаплотипа: первый включал гены и аллели – Lpx-B1.3+Lpx-B1.b, второй – LpxB1.2+LpxB1.1a и третий – LpxB1.2+LpxB1.1c. Эти гаплотипы демон- стрировали, соответственно, высокий, средний и низкий уровни функциональных транскриптов Lpx-B1 и фермен- тативной активности в созревшем зерне.

Известны коммерческие сорта-носители разных ге- нов, Lpx-B1.1, Lpx-B1.2, Lpx-B1.3, и аллелей в локусе Lpx-B1.1. В частности, сорта Kofa и Aureo содержат аллель Lpx-B1.1c. Описаны возможности накопления каротиноидов и генетика липоксигеназы у сортов: Primadur (имеет высокое содержание каротиноидов и высокую активность липоксигеназы), Cosmodur (высокая концентрация каротиноидов, низкая активность липоксигеназы – Lpx-B1.1с), Trinakria (низкая концентрация каротинои- дов, высокая активность липоксигеназы), Creso (низкое содержание каротиноидов и низкая активность липокси- геназы – Lpx-B1.1с) (De Simone et al., 2010). По данным A. Verlotta с коллегами (2010) в табл. 2 показано рас- пределение сортов разных периодов селекции (до 1971 г. и в 1971–2005 гг.) по их принадлежности к различным гаплотипам. Вызывает внимание увеличение частоты встречаемости второго и третьего гаплотипов в сортах последних периодов селекции. Необходимо подчеркнуть, что этот результат достигнут селекционерами без по- нимания и учета в селекционных процедурах генетики окислительных процессов в зерне.

**Table 2.**
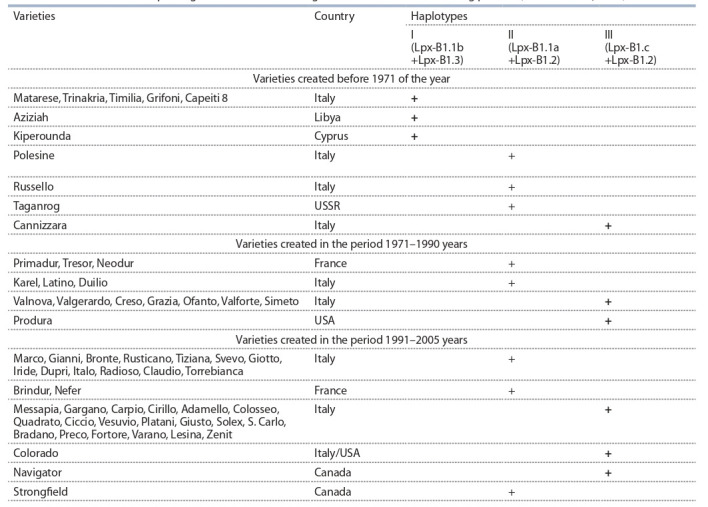
Distribution of the Lpx-B1 genes and alleles among cultivars of different breeding periods (Verlotta et al., 2010) Note. The “+” sign indicates the belonging to the haplotype.

Второй по значимости воздействия на цвет конечных продуктов катаболический фермент – полифенолоксида- за (РРО). Активность фермента контролируют два гена, идентифицированных на гомеологичных хромосомах второй группы, 2А и 2В (Jimenez, Dubcovsky, 1999; Simeone et al., 2002; Watanabe et al., 2004, 2006). В частности, R. Simeone с коллегами (2002) сообщили о значительном генетическом эффекте на активность РРО локуса на длин- ном плече 2А хромосомы. Гены Тс1 и Тс2 в дистальных частях 2AL и 2BL на расстоянии 46.08 и 40.7 сМ от цен- тромеры картированы N. Watanabe с коллегами (2004). Изучение RIL, полученной от скрещивания сортов Jennah Khetifa и Cham 1, показало, что локус в хромосоме 2А обеспечивал 49.1 % активности РРО, низкая активность сегрегировала с молекулярным маркером Xgwm312@2A (Watanabe et al., 2006).

Два гомологичных семейства РРО были картированы по второй гомеологичной группе хромосом и названы РРО-1 (РРО-A1 и РРО-В1) и РРО-2 (РРО-А2 и РРО-В2) (Beecher et al., 2012). Использование маркера, специфич- ного для мягкой пшеницы (РРО18), для анализа твердой пшеницы позволило обнаружить четыре аллеля РРО-А1: РРО-А1b, PPO-A1f, PPO-A1e, PPO-A1g (He et al., 2009). Используя 111 образцов твердой пшеницы, F. Taranto с коллегами (2012) определили связь различных аллелей РРО-А1 с уровнем активности фермента. Аллель РРО-А1f был связан с высокой, тогда как РРО-А1b и РРО-А1g – с низкой активностью фермента. Эти ученые разработали также новый маркер (MG18), способный обнаружить те же аллели, что и при помощи маркера РРО18, но более эффективно и с более низкой вариабельностью актив- ности РРО внутри каждой группы сортов, несущих один и тот же аллель (Taranto et al., 2012). В маркер-ассоции- рованной селекции с целью снижения активности РРО целесообразно использовать и паралогичные гены РРО-В1 и РРО-В2 с применением соответствующих маркеров MG08 и MG33, предложенных F. Taranto с коллегами (2015). Гены PPO-B1 и PPO-B2 были расположены на расстоянии 11.4 сМ от центромеры на хромосоме 2BL. Скрининг коллекции твердой пшеницы с помощью мар- керов MG08 и MG33 позволил идентифицировать четыре и два аллеля соответственно, включая три новых аллеля гена PPO-B1: PPO- B1b, PPO-B1c и PPO-B1d, и один но- вый аллель PPO-B2 – PPO-B2d. Маркер MG33 способен распознавать два аллеля РРО-В2, связанных с высокой (РРО-В2d) и низкой (РРО-В2а) активностью фермента (Taranto et al., 2015).

Значительное влияние на признаки YI, YPC и цвет ма- каронных изделий оказывает альдегидоксидаза (АО; ЕС 1.2.3.1) (Colasuonno et al., 2017b). Три изоформы фермента пшеницы, АО1, АО2, АО3, были локализованы на 2-, 5-, 7-хромосомных группах соответственно. Третий ген альдегидоксидазы, АО-А3, расположенный на хромосо- ме 7AL, связан с QTL, влияющим на признаки YI и YPC (Colasuonno et al., 2017b). Эксперименты с двумя сортами, Cicco (низкое содержание каротиноидов в зерне) и Svevo (высокое содержание каротиноидов), с применением метода qRT-PCR выявили высокий уровень экспрессии гена АО-АЗ у первого сорта и низкий у второго, что подтверждает отрицательный эффект продуктов гена в период накопления каротиноидов. На основе SNP данных, со- ответствующих изоформе фермента, на хромосоме 7AL картирован маркер IWB59875, который предложен для процедур маркерной селекции для повышения содержа- ния каротиноидов в зерне и цвета макаронных изделий (Colasuonno et al., 2017b).

Пероксидазы – ферменты, катализирующие общую реакцию: ROOH + H_2_O_2_ = ROH + H_2_O + 1/2O_2_ (Feillet et al., 2000). Паста, произведенная из зерна сортов с высоким уровнем активности пероксидазы, имеет буро-корич- невый цвет и низкие потребительские качества (Sisson, 2008). В то же время в процессе изготовления макарон пероксидаза не проявляется в связи с недоступностью перекиси водорода (Ficco et al., 2014). Активность фер- ментов этой группы в зерне твердой пшеницы значительно меньше, чем в зерне мягкой пшеницы. В связи с этим большинство исследований по их изучению проведено на мягкой пшенице. В литературе отсутствуют сведения о специфических маркерах в геноме твердой пшеницы, связанных с QTL или генами низкой активности. В ряде публикаций отмечено, что гены пероксидазы расположе- ны в гомеологичных хромосомах групп 1, 2, 3, 4 и 7 (Liu et al., 1990; Wei et al., 2015). В зерне твердой пшеницы установлено функционирование 12 изоформ перокси- дазы, различающихся по активности в период налива, созревания и прорастания зерна. Некоторые изоформы имеют специфическую локализацию в зерне – перикарп, эндосперм, зародыш. Наиболее важная изоформа, Р-5, расположена в эндосперме и оказывает значимое влияние на потемнение (коричневатость) макаронных изделий (Feillet et al., 2000). При использовании полногеномного секвенирования и нулли-тетрасомных линий сорта Чайниз Спринг обнаружены и локализованы два гена, TaPod-A2 и TaPod-D1, на хромосомах 7AS и 7DS. Анализ SNP выявил для двух аллелей локуса TaPod-D1 два функциональ- ных маркера, POD-7D1 и POD-7D6, с высокой и низкой активностью пероксидазы соответственно (Geng et al., 2019). Эти данные, полученные на мягкой пшенице, с учетом ортологичности геномов А и В в перспективе можно адаптировать к проблемам маркерной селекции твердой пшеницы. В отечественной литературе известна публикация А.А. Вьюшкова (2004), в которой приведены значительные сортовые различия твердой пшеницы по активности пероксидазы в крупке (эндосперме).

## Результаты и перспективы применения MAS в селекции твердой пшеницы

Несмотря на большое количество работ по локализации и маркированию QTL, связанных с высокой концентрацией каротиноидов в зерне и крупке, результаты прямой про- верки применения MAS (селекция с помощью маркеров) представлены ограниченно. Patil с коллегами (2018) со- общили о высокой эффективности использования маркера Psy-A1SSRe, сцепленного с QTL и локусом Psy-A1, на хромосоме 7AL. Маркер Psy-1SSR разработан на основе вариаций в промоторной области PSY-1, он позволяет идентифицировать восемь аллелей Psy-A1 и семь аллелей Psy-B1 одновременно. Маркер Psy-A1SSRe, расположен- ный в 7AL в большом QTL для YPC, вместе с ранее уста- новленным маркером Qyp.macs-7A был идентифицирован в популяции RIL-PDW233/Bhalegaon 4. Родительский сорт PDW 233 в этой популяции является носителем QTL высокой концентрации пигментов. Эти маркеры были применены для улучшения индийских сортов MACS 3125 и HI8498 с низкой концентрацией пигментов (3.57 и 3.26 ppm соответственно), которые были взяты в качестве рекуррентных родителей в скрещиваниях с PDW 233 – донором QTL для YPC с высокой концентрацией пиг- ментов (8.36 ppm). Селекционные линии, полученные с применением методов MAS на основе MACS 3125 и HI 8498, показали значительное увеличение YPC: 6.16–7.7 и 5.0–7.46 ppm соответственно. В настоящее время MAS используется в CIMMYT и в Канаде для отбора селекци- онных линий с низкой активностью липоксигеназы при помощи маркера LOXA, нацеленного на аллель Lpx-B1.1c (Randhawa et al., 2013; Dreisigacker et al., 2016; N’Diaye et al., 2017, 2018).

В России во всех лабораториях, осуществляющих селекцию на увеличение концентрации каротиноидов в зерне, индекса желтизны семолины и конечных продуктов, применяются методы традиционной селекции. За период научной селекции и особенно за последние 30 лет эти признаки были улучшены. Сорта, созданные на первых этапах, – Мелянопус 69, Гордеиформе 432, Мелянопус 26, Гордеиформе 179, Гордеиформе 675, накапливают в зерне 3.6–5.0 ppm каротиноидных пигментов. Сорта, широко возделывавшиеся в 60-80-х годах ХХ в., – Безенчук- ская 105, Харьковская 46, Безенчукская 139, – превышают этот уровень незначительно (~5 %) (Мясникова и др., 2019). Положительные изменения наблюдались у сортов Светлана (1987 – год включения в реестр) и Саратовская золотистая (1993), которые накапливали 6.0–7.0 ppm пигментов в зерне (Васильчук, 2001). Среди современных сортов яровой твердой пшеницы заметно выделяются по содержанию каротиноидов в зерне Безенчукская золоти- стая (8.5–9.0 ppm) и Безенчукская крепость (7.5–8.5 ppm). Весь набор современных сортов образует непрерывный ряд изменчивости с шагом в 10–15 % и разницей между крайними вариантами в 200 % (Безенчукская золотистая – Алтайская нива). Изучение отечественных сортов разных этапов селекции в одиннадцати экологических средах позволило установить, что фенотипическое варьирование признака определяется генотипической и средовой вариансами с незначительными эффектами генотип-средовых взаимодействий. По результатам кластерного анализа параметров адаптивности, стабильности и отзывчивости, сорта отчетливо распределялись на кластеры. В группу с оптимальным сочетанием величины, стабильности и отзывчивости признака вошли Безенчукская золотистая, Безенчукская крепость, Безенчукская 210, Саратовская зо- лотистая. Эти генотипы рекомендуется использовать для создания рекомбинантных инбредных линий с целью мар- кирования QTL, контролирующих синтез каротиноидов в зерне твердой пшеницы, и организации на этой основе маркер-опосредованной технологии селекции (Мясникова и др., 2019).

## Заключение

Результаты исследований, рассмотренные в этой статье, показывают реализованные за последние двадцать лет цели в понимании биохимических, генетических ме- ханизмов регулирования метаболизма каротиноидных пигментов в твердой пшенице. Данные, полученные на разнообразном растительном материале с применением современных классов ДНК-маркеров, и согласованные в разнообразных экспериментах генетические карты по- зволили выявить наиболее важные гены, участвующие в контроле биосинтеза, накопления и катаболизма кароти- ноидов. Наиболее изученными и подтвержденными в ряде экспериментов QTL являются те, которые расположены на хромосомах 3AS (связаны с геном LCYE), 7AL и 7BL (оба тесно связаны с аллелями PSY 1). Перспективно для MAS применение маркера LOXA, нацеленного на аллель Lpx-B1.1c для снижения активности липоксиге- назы. Технология MAS с использованием известных для этих QTL маркеров отличается от методов традиционной фенотипической селекции высокой производительностью и эффективностью. В то же время в ряде исследований были представлены данные о том, что большинство QTL не могут быть широко использованы в процедурах MAS, что связано с уровнем их валидации в фенотипических соотношениях в локально адаптированных группах се- лекционного материала (Pozniak et al., 2012).

Другие QTL, представленные и охарактеризованные в обзоре, требуют дополнительных исследований для идентификации генов-кандидатов, участвующих в накоплении/ деградации каротиноидов. В перспективе можно ожидать широкое внедрение методов геномной селекции в программы улучшения цвета конечных продуктов из твердой пшеницы. С учетом аддитивных эффектов генов, контролирующих уровень пигментов и превалирование генотипа признака над средой, применение методов геномной селекции позволит ускорить селекционный про- цесс на основе не только молекулярной идентификации функционирующих в популяции необходимых QTL, но и целенаправленного получения трансгрессий в результате пирамидирования различных генов. В связи с этим даль- нейший акцент в селекционно-генетических мероприя- тиях будет сделан на анализе генетической изменчивости в коллекциях зародышевой плазмы твердой пшеницы и мутантных популяциях. Перспективны методы редакти- рования генома с использованием CRIS-Cas9, если их применять, исходя из понимания функций гомеологичных генов с аддитивным эффектом (Patil et al., 2018). Характе- ристика каждого гена позволит вырабатывать стратегии диверсификации генетической системы каротиноидных пигментов и расширить существующие вариации, до- ступные селекционерам.

Непосредственное применение маркеров QTL, апробированных на растительном материале зарубежных стран в России в процедурах MAS и геномной селекции, воз- можно, если в качестве доноров использовать сорта за- рубежной селекции, несущие соответствующие QTL и маркеры. При этом маркеры должны быть эффектив- ными в экологических зонах российских селекционных центров. Однако и в этом случае могут быть проблемы преодоления недостаточной адаптивности привлекаемых в качестве исходного материала генотипов. Селекция на увеличение концентрации пигментов в зерне и продуктах его переработки в России, безусловно, может использо- вать генетический материал и маркеры, разработанные в иностранных центрах, но базироваться она должна на отечественном исходном материале и адаптированных к нему технологиях маркер-опосредованной селекции.

## Conflict of interest

The authors declare no conflict of interest.
